# Clinical utility of and correlation between Sniffin' Sticks and TIB smell identification test (TIBSIT) among Hong Kong Chinese with or without chronic rhinosinusitis

**DOI:** 10.3389/falgy.2024.1292342

**Published:** 2024-01-24

**Authors:** Hugo W. F. Mak, Shi Yeung Ho, Jane C. Y. Wong, Valerie Chiang, Elaine Lee, Jackie S. H. Yim, Birgitta Y. H. Wong, Philip H. Li

**Affiliations:** ^1^ Division of Rheumatology and Clinical Immunology, Department of Medicine, Queen Mary Hospital, The University of Hong Kong, Hong Kong, Hong Kong SAR, China; ^2^ Department of Ear, Nose and Throat, Queen Mary Hospital, Hong Kong, Hong Kong SAR, China; ^3^ Division of Clinical Immunology, Department of Pathology, Queen Mary Hospital, Hong Kong, Hong Kong SAR, China

**Keywords:** smell, olfactory dysfunction, utility, chronic rhinosinusitis, Hong Kong, Chinese

## Abstract

**Introduction:**

Olfactory dysfunction (OD) is common among patients with chronic rhinosinusitis (CRS). Validated and culturally specific tests, such as the “Sniffin’ Sticks” test (SST) and the TIB Smell Identification Test (TIBSIT), are crucial for the diagnosis and monitoring of OD. However, they have not been utilised in Hong Kong Chinese and their correlations are unknown.

**Methods:**

Twelve CRS patients and twenty healthy volunteers were prospectively recruited from a joint allergy-otorhinolaryngology clinic in Hong Kong and performed both SST and TIBSIT. Demographics, baseline characteristics and all test results were compared and analysed.

**Results:**

Patients with CRS demonstrated significantly lower test scores than healthy controls (all *p* < 0.001). Significant and strong correlations were observed between all composite and subtest scores, particularly between the composite SST and TIBSIT scores (*ρ* = 0.789, *p* < 0.001). Multivariate analysis demonstrated that the presence of CRS and increasing age were significantly associated with OD.

**Conclusion:**

Both SST and TIBSIT are useful olfactory tests and are strongly correlated among Hong Kong Chinese. We advocate that either test can be used for measuring OD among CRS patients.

## Introduction

Olfactory dysfunction (OD), characterized by “partial or complete smell loss” (hyposmia and anosmia respectively), or “qualitative dysfunction of smell in the presence or absence of an odour object” (parosmia and phantosmia respectively), is a common symptom associated with significantly impaired quality of life and affects activities of daily living (1, 2). Patients suffering from various allergic conditions, most notably inflammatory rhinopathologies such as chronic rhinosinusitis (CRS), are especially prone to develop OD (3, 4). The prevalence of OD has been reported to be within the range of 30%–78% among CRS patients, with varying rates depending on the tests used for measuring OD (5). In addition to genuine inter-population differences, this vast variation in prevalence rates thus far reported is likely contributed by the lack of access to testing facilities and under-reporting in many regions of the world (6–8). Therefore, more standardised and validated tests for olfactory function would be of substantial value towards both research and clinical management of patients suffering from OD.

For this sake, a number of smelling tests were developed in the past few decades to allow a more objective olfactory evaluation. For instance, respectively in 1984 and 1997, the University of Pennsylvania Smell Identification Test (UPSIT) and the “Sniffin’ Sticks” test (SST) (Burghart Messtechnik, Wedel, Germany) were developed in the United States and Germany as semi-objective, psychophysical assessment tools for olfactory function (9, 10). Since their development, UPSIT and SST have gained popularity especially among ear, nose and throat (ENT) surgeons. The commercially available UPSIT and SST have also been validated in various populations and has become one of the more widely applied tests for OD, especially in North American and European countries (11–23). However, available olfactory tests remained limited beyond Western populations. Subsequently, a Taiwanese group developed and validated a brief, office-based screening test for OD known as the TIB Smell Identification Test (TIBSIT) (Top International Biotech, Taipei, Taiwan) in 2015 (24, 25). At the time of writing, application and validation of TIBSIT has been limited to Taiwan and Malaysia (25–27). To the best of our knowledge, neither SST nor TIBSIT have been utilised among Hong Kong Chinese. Furthermore, the correlation (if any) between SST and TIBSIT is unknown. Of note, both SST and TIBSIT encompass odour identification (i.e., recognition of daily encountered odours) which is dependent on patients' familiarization with the tested odours and is thus highly culturally specific. Therefore, it is important for both SST and TIBSIT to be applied and tested among culturally specific populations, especially in cultures for whom these tests were not initially designed. In this study, we aim to explore the clinical utility of both tests among patients with CRS and healthy volunteers in Hong Kong, as well as examine the association between SST (and its subtests) and TIBSIT.

## Methods

### Study participants

Consecutive patients attending a joint allergy-ENT clinic with newly diagnosed CRS were prospectively recruited at Queen Mary Hospital in Hong Kong between January 2022 and June 2022. For all patients, the diagnosis of CRS was confirmed with the exclusion of other conditions that may contribute to OD by joint clinical assessment by both allergists and otorhinolaryngologists with nasoendoscopy assessment. Twenty healthy individuals, who reported having a normal sense of smell and no past medical history of smelling disorders or conditions related to OD, were also recruited as controls. Only adults (individuals of at least 18 years old) were included. Individuals with nasal tumours, history of relevant trauma, neurological disorders, recent upper respiratory tract/SARS-CoV-2 infection or concomitant nasal pathologies were excluded. Baseline demographic data was also collected to study the effect of these demographic factors on smelling function. This study was approved by the Institutional Review Board of the University of Hong Kong/Hospital Authority Hong Kong West Cluster. All participants gave informed consent.

### Instruments

Under the supervision of the attending Allergist, all participants were assessed by SST and TIBSIT performed in a well-ventilated room as per product manual by trained allergy nurses, who underwent online training offered by the manufacturer. SST is a nasal chemosensory test in which pen-like, odourant-containing sticks are presented to individuals. It is composed of three subtests, namely odour threshold (T), odour discrimination (D) and odour identification (I). In the T test, patients are presented with different concentrations (16 levels) of n-butanol, and their olfactory sensitivities are assessed using a repeated staircase approach, in which multiple turning points are determined and averaged to yield an overall T score. The D test uses 16 triplets of sticks, whereof two share the same odourant, differentiating from the target stick. Individuals' D scores are assigned based on the number of times they correctly identify the different smelling sticks. The I test uses 16 sticks containing a variety of everyday smells. In each round, four options are given to individuals, who are required to identify the presented odour from three other distractors. I scores equate to the number of correctly selected odours. Both D and I tests require the individual to be blindfolded i.e., are single-blinded tests. Throughout the entire test, individuals are also required to choose an option even if they cannot give a confident response (forced-choice). The respective scores from each of the three subtests are aggregated for a composite TDI score for interpretation. Hyposmia and functional anosmia are defined as TDI score ≤ 30.5 and ≤ 16 respectively. Supersmellers refers to those reaching the highest decile score in the 21–30 age group (TDI score ≥ 41.5) (28).

TIBSIT is a smelling test recently developed for Taiwanese (ethnically Chinese), who likely share a similar cultural background, including dietary habits, with Hong Kong Chinese. TIBSIT requires individuals to identify 8 odours common to Taiwanese Chinese and each odour is presented twice, giving rise to a total of 16 questions. Unlike SST, TIBSIT uses a “scratch-and-smell” design. Tested individuals are given a disposable test booklet, which contains a scratchable test strip with embedded fragrant microcapsules on each page. They are instructed to scratch the strip surface then identify the odour among 4 options (forced choices) and state their confidence in identification (1: not detectable; 2: detectable, but not sure; 3: detectable). Scores from all the questions are then summated and interpreted as a composite TIBSIT score.

### Statistical analysis

Continuous variables were expressed in median (lower quartile—upper quartile) and categorical variables were expressed as number (percentage). All statistical analyses were performed on IBM SPSS Statistics version 28.0 (IBM Co., Armonk, NY, USA). Continuous and categorical variables were compared between the healthy control and the patient group with Mann–Whitney *U*-Test and Chi-square test respectively. Correlations of scores of different olfactory tests (and subtests) were assessed by Spearman correlation. We defined weak, moderate, and strong correlations as 0 < *ρ* ≤ 0.39, 0.40 ≤ *ρ* ≤ 0.59 and *ρ* ≥ 0.60 respectively (29, 30). We also examined the effect, if any, of age and sex on olfactory test scores. Variables with *P*-value < 0.1 in univariate analysis (Spearman correlation for age and Mann–Whitney *U*-test for sex) were included in subsequent multivariate linear regression analysis. Line plots were prepared using R version 4.3.1 (R Foundation, Vienna, Austria) (31). Two-sided *P*-value < 0.05 indicates statistical significance.

## Results

In total, 32 individuals were included in this study (12 CRS patients and 20 healthy volunteers). All participants were Han Chinese, 43.8% (14/32) were males and the median age was 53.5 (interquartile range: 45.0–59.5) years. There were no significant demographic differences between patients and controls (Table 1). Olfactory test scores of all participants are shown in Table 2. Compared to healthy controls, CRS patients demonstrated significantly lower scores in all conducted tests and subtests. Overall, the median composite TDI score of CRS patients was 13.5, compared to 34.4 for healthy controls (*p* < 0.001). Respectively 33.3% (4/12) and 58.3% (7/12) of CRS patients were found to have hyposmia and functional anosmia, the overall OD prevalence in this CRS cohort was thus 91.7% (11/12), contrasting the control group where only two individuals (10.0%), who aged 88 and 54, were within the range of hyposmia (*p* < 0.001). A significant difference was found using TIBSIT as well (median score: 0.0 vs. 41.5, *p* < 0.001).

**Table 1:** Demographic characteristics of healthy controls and chronic rhinosinusitis patients.

	Total *N* = 32	Healthy controls *N* = 20	CRS patients *N* = 12	*p*-value
Age (years)	53.5 (45.0–59.5)	52.5 (41.0–60.3)	57.0 (47.0–59.5)	0.239
Male sex	14 (43.8)	8 (40.0)	6 (50.0)	0.581
Ethnic Chinese	32 (100.0)	20 (100.0)	12 (100.0)	N/A

CRS, chronic rhinosinusitis.

**Table 2:** Olfactory test scores of healthy controls and chronic rhinosinusitis patients.

	Total *N* = 32	Healthy controls *N* = 20	CRS patients *N* = 12	*p*-value
T score (SST)	10.5 (2.2–12.0)	11.6 (10.4–12.2)	1.0 (1.0–7.1)	* < 0.001
D score (SST)	10.5 (7.0–12.0)	11.0 (10.0–12.0)	6.5 (4.3–7.8)	* < 0.001
I score (SST)	10.0 (7.0–13.0)	12.0 (9.3–13.8)	6.0 (4.3–8.5)	* < 0.001
TDI score (SST)	31.0 (18.4–35.9)	34.4 (31.1–36.9)	13.5 (10.3–27.2)	* < 0.001
Hyposmic or anosmic (SST)	13 (40.6)	2 (10.0)	11 (91.7)	* < 0.001
TIBSIT score	39.0 (9.5–44.0)	41.5 (39.3–47.5)	0.0 (0.0–23.5)	* < 0.001

Bold denotes statistical significance.

CRS, chronic rhinosinusitis; SST, “Sniffin’ Sticks” test; TIBSIT, TIB smell identification test; T, threshold; D, discrimination; I, identification; TDI, composite of T, D and I scores.

**Figure 1:**
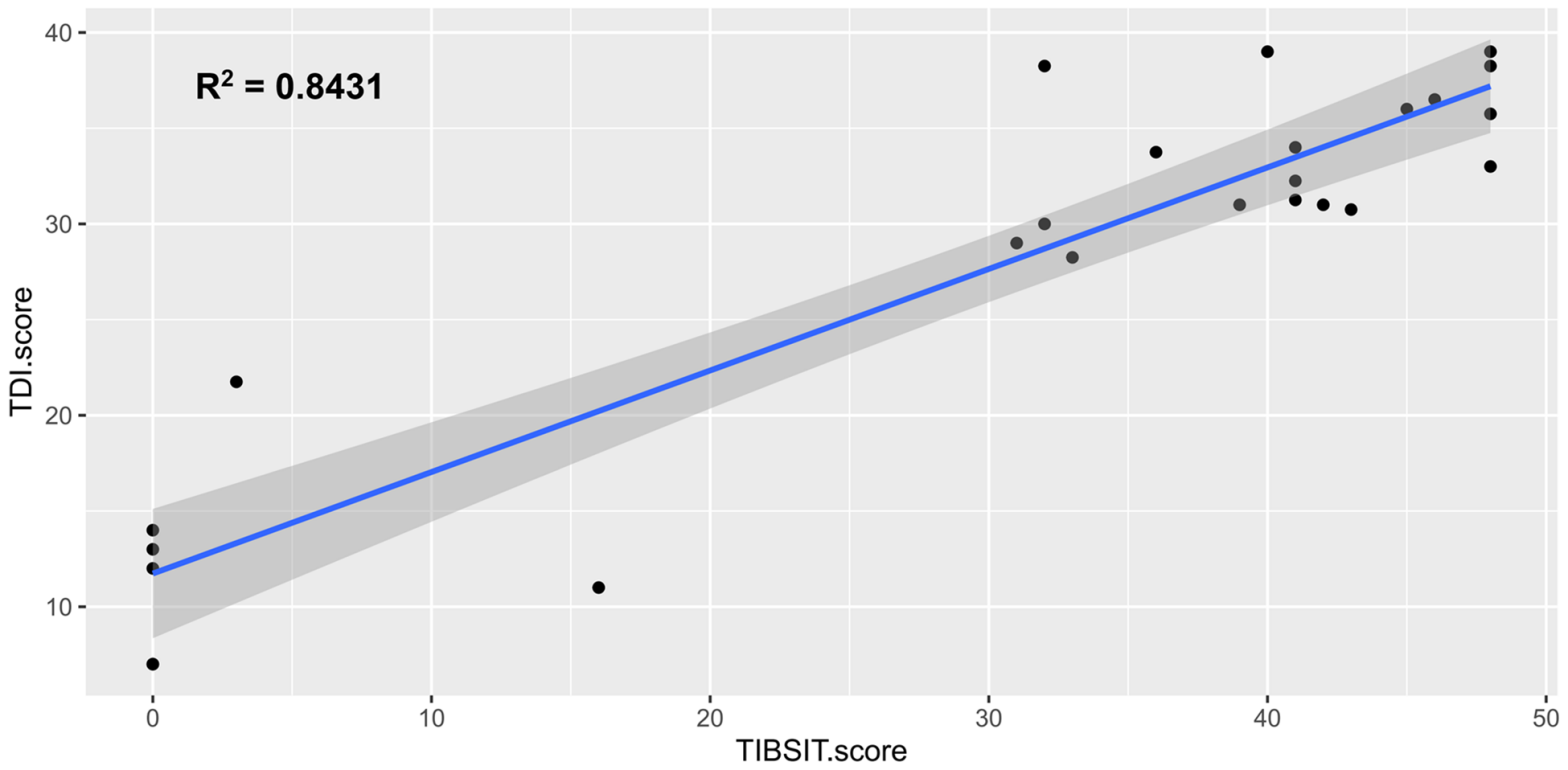
Correlation between TDI score (SST) and TIBSIT score. TDI, composite of threshold, discrimination and identification scores; TIBSIT, TIB smell identification test.

Association analysis demonstrated that all tests and subtests were moderately or strongly correlated between each other (all *ρ* > 0.4 and *p* < 0.001). A matrix detailing all the correlation coefficients is shown in Table 3. Specifically, there was a particularly strong correlation between TDI (SST) and TIBSIT scores (*ρ* = 0.789, *p* < 0.001; Figure 1). Individual subtests in SST also carried strong correlations to TIBSIT.

**Table 3:** Correlation matrix of olfactory test scores and age.

	Age	T score (SST)	D score (SST)	I score (SST)	TDI score (SST)	TIBSIT score
Age	–	−0.338^a^	−0.446*	−0.301^a^	−0.428*	−0.553**
T score (SST)		–	0.459**	0.476**	0.707***	0.746***
D score (SST)			–	0.591***	0.782***	0.661***
I score (SST)				–	0.843***	0.659***
TDI score (SST)					–	0.789***
TIBSIT score						–

TIBSIT, TIB smell identification test; T, threshold; D, discrimination; I, identification; TDI, composite of T, D and I scores.

* *p* < 0.05.

** *p* < 0.01.

*** *p* < 0.001.

^a^ *p* < 0.1 but >0.05.

**Figure 2:**
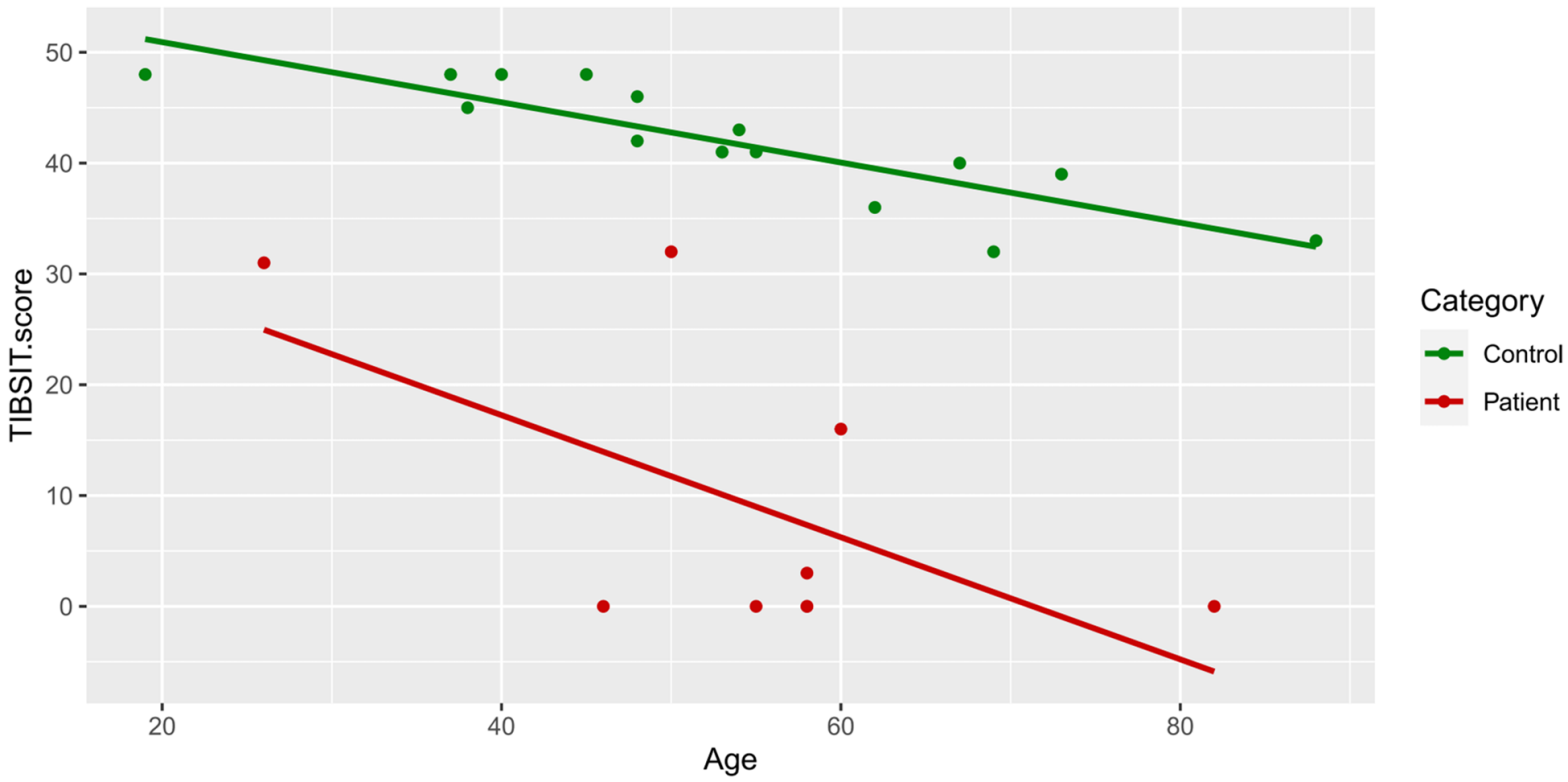
TIBSIT scores of healthy controls and chronic rhinosinusitis patients against age. TDI, composite of threshold, discrimination and identification scores; TIBSIT, TIB smell identification test.

In univariate analysis, older age was moderately associated with lower D, TDI and TIBSIT scores (*r* > 0.4 and *p* < 0.05), and correlations with T and I scores also reached near-significance for multivariate analysis (Table 3 and Figure 2). As shown in Supplementary Figure S1, females scored numerically higher in TIBSIT and SST subsets, but statistical significance was only attained in the T score (*p* = 0.039). In the linear regression model, when the presence of CRS, age and sex were considered, CRS remained a significant factor in all tests and subtests (all *p* < 0.001; Supplementary Table S1).

## Discussion

This study applies and reports the association between two different olfactory tests, SST and TIBSIT, among Hong Kong Chinese. Using either SST or TIBSIT, our cohort of CRS patients demonstrated significant OD with the majority of our cohort found to be hyposmic or anosmic. All subtest and composite test scores were significantly inferior to healthy controls, demonstrating the clinical utility of SST and TIBSIT. This provides a basis for the clinical application of the two tests in the screening and diagnosis of OD in Hong Kong Chinese, as well as monitoring of disease progression and response to treatment. In SST, our cohort obtained a particularly low T test score (median score of 1), corroborating previous reports of T scores generally being lower than D and I scores among CRS patients (5). Further studies are required to differentiate genuine differences in impairment of detecting odour thresholds (vs. discrimination or identification), rather than inherent differences between SST subtests or reporting.

Utilisation and cross-cultural validation of olfactory tests have only been performed in selected populations, especially beyond Western cohorts (14, 32, 33). Prior to this study, results from SST and TIBSIT cannot yet be used interchangeably and results between studies using different tests cannot be readily compared. Interestingly, we found that SST and TIBSIT demonstrated strong correlations among Hong Kong Chinese despite initially being developed for vastly different populations. This may be due to Hong Kong being a culturally diverse territory, with locals previously exposed to and able to recognize a wide variety of odourants commonly found in both Eastern and Western cultures. We therefore propose either SST or TIBSIT to be used among Hong Kong Chinese, and it would be of interest to evaluate whether this phenomenon also exists in other culturally diverse populations. Such studies would be of particular interest in the Asia-Pacific region, especially given its rapidly expanding disease burden of allergic diseases (such as CRS), coupled with distinctive intra- and inter-regional variations as well as disparities in access to allergy care (34–41).

Consistent with previous studies, we also identified a decline in olfactory function with increasing age among both CRS patients and healthy individuals of our cohort (18, 25, 26). This is likely related to the natural degeneration of the olfactory system (such as the olfactory neuroepithelium and bulb) with increasing age, leading to progressive OD and inability to discriminate between odours (42, 43). Indeed, even among healthy controls, we did identify two tested subjects who were hyposmic, which we believe to be physiological. Conversely, although we also found that females tended to perform better in olfactory tests as reported by previous studies, this did not reach statistical significance (20, 28, 43, 44). Whether this non-significance was due to limitations in study design, cross-cultural or genuine inter-population differences will require future multi-ethnic studies. Overall, our results are largely reminiscent of prior studies.

There were several limitations to this study. For example, we had a relatively small sample size which may not be sufficient to accurately reflect the normative values of our population. Future large-scale studies are needed to establish the population norms in Hong Kong. Further dedicated studies to delineate other properties of these and other smelling tests e.g., test-retest reliability are also warranted. Although all patients were screened by both allergists and ENT specialists, detailed clinical information or possible confounders such as educational background or history of pregnancy were not recorded (44). For female patients, information regarding their hormonal status, such as use of oral contraceptives or hormonal therapy, which is reported to positively influence olfactory test performance, were not available (45). There also exists possible referral bias as the joint allergy-ENT clinic primarily receives referrals for more severe CRS cases which warrant specialist care. This may lead to an overestimation of the prevalence and burden of OD among CRS patients in Hong Kong.

In summary, both SST and TIBSIT are useful instruments for OD assessments among Hong Kong Chinese. The two tests are strongly correlated and we advocate that either test can be used to evaluate OD among CRS patients. Increasing age, and possibly male sex were associated with poorer performance in smelling tests.

## Data Availability

The raw data supporting the conclusions of this article will be made available by the authors, without undue reservation.

## References

[1] Patel ZM Holbrook EH Turner JH Adappa ND Albers MW Altundag A International consensus statement on allergy and rhinology: olfaction. Int Forum Allergy Rhinol. (2022) 12(4):327–680. 10.1002/alr.22929 35373533 PMC12261282

[2] Miwa T Furukawa M Tsukatani T Costanzo RM DiNardo LJ Reiter ER. Impact of olfactory impairment on quality of life and disability. Arch Otolaryngol Head Neck Surg. (2001) 127(5):497–503. 10.1001/archotol.127.5.497 11346423

[3] Macchi A Giorli A Cantone E Carlotta Pipolo G Arnone F Barbone U Sense of smell in chronic rhinosinusitis: a multicentric study on 811 patients. Front Allergy. (2023) 4:1083964. 10.3389/falgy.2023.1083964 37152304 PMC10160403

[4] James J Palte IC Vilarello BJ Axiotakis LG Jr Jacobson PT Gudis DA Beyond Aroma: a scoping review on the impact of chronic rhinosinusitis on retronasal olfaction. Front Allergy (2022) 3:969368. 10.3389/falgy.2022.969368 36118172 PMC9470759

[5] Kohli P Naik AN Harruff EE Nguyen SA Schlosser RJ Soler ZM. The prevalence of olfactory dysfunction in chronic rhinosinusitis. Laryngoscope. (2017) 127(2):309–20. 10.1002/lary.26316 27873345 PMC5258829

[6] Hui HKS Li TS Lo WLW Kan AKC Ho SY Yeung WYW Sensitisation profile of Chinese allergic rhinitis patients and effectiveness of a joint allergy-ent clinic. Allergo J Int. (2023) 32(2):29–37. 10.1007/s40629-022-00218-5 35822075 PMC9261891

[7] Mullol J Marino-Sanchez F Valls M Alobid I Marin C. The sense of smell in chronic rhinosinusitis. J Allergy Clin Immunol. (2020) 145(3):773–6. 10.1016/j.jaci.2020.01.024 32145875

[8] Hummel T Whitcroft KL Andrews P Altundag A Cinghi C Costanzo RM Position paper on olfactory dysfunction. Rhinology. (2016) 56(1):1–30. 10.4193/Rhino16.248 28623665

[9] Doty RL Shaman P Dann M. Development of the university of Pennsylvania smell identification test: a standardized microencapsulated test of olfactory function. Physiol Behav. (1984) 32(3):489–502. 10.1016/0031-9384(84)90269-5 6463130

[10] Hummel T Sekinger B Wolf SR Pauli E Kobal G. ’Sniffin’ Sticks’: olfactory performance assessed by the combined testing of odor identification, odor discrimination and olfactory threshold. Chem Senses. (1997) 22(1):39–52. 10.1093/chemse/22.1.39 9056084

[11] Ogihara H Kobayashi M Nishida K Kitano M Takeuchi K. Applicability of the cross-culturally modified university of Pennsylvania smell identification test in a Japanese population. Am J Rhinol Allergy. (2011) 25(6):404–10. 10.2500/ajra.2011.25.3658 22185745

[12] Jiang RS Kuo LT Wu SH Su MC Liang KL. Validation of the applicability of the traditional Chinese version of the University of Pennsylvania smell identification test in patients with chronic rhinosinusitis. Allergy Rhinol (Providence). (2014) 5(1):28–35. 10.2500/ar.2014.5.0084 25199144 PMC4019742

[13] Altundag A Tekeli H Salihoglu M Cayonu M Yasar H Kendirli MT Cross-Culturally modified University of Pennsylvania smell identification test for a Turkish population. Am J Rhinol Allergy. (2015) 29(5):e138–41. 10.2500/ajra.2015.29.4212 26358338

[14] Shu CH Yuan BC Lin SH Lin CZ. Cross-cultural application of the “Sniffin’ Sticks” odor identification test. Am J Rhinol. (2007) 21(5):570–3. 10.2500/ajr.2007.21.3075 17999792

[15] Shu CH Yuan BC. Assessment of odor identification function in Asia using a modified “Sniffin’ Stick” odor identification test. Eur Arch Otorhinolaryngol. (2008) 265(7):787–90. 10.1007/s00405-007-0551-2 18064480

[16] Yuan BC Lee PL Lee YL Lin SH Shu CH. Investigation of the Sniffin’ Sticks olfactory test in Taiwan and comparison with different continents. J Chin Med Assoc. (2010) 73(9):483–6. 10.1016/S1726-4901(10)70103-9 20875622

[17] Konstantinidis I Printza A Genetzaki S Mamali K Kekes G Constantinidis J. Cultural adaptation of an olfactory identification test: the Greek version of Sniffin’ Sticks. Rhinology. (2008) 46(4):292–6. 19145999

[18] Neumann C Tsioulos K Merkonidis C Salam M Clark A Philpott C. Validation study of the “Sniffin’ Sticks” olfactory test in a British population: a preliminary communication. Clin Otolaryngol. (2012) 37(1):23–7. 10.1111/j.1749-4486.2012.02431.x 22433135

[19] Fjaeldstad A Kjaergaard T Van Hartevelt TJ Moeller A Kringelbach ML Ovesen T. Olfactory screening: validation of Sniffin’ Sticks in Denmark. Clin Otolaryngol. (2015) 40(6):545–50. 10.1111/coa.12405 25721152

[20] Ribeiro JC Simoes J Silva F Silva ED Hummel C Hummel T Cultural adaptation of the Portuguese version of the “Sniffin’ Sticks” smell test: reliability, validity, and normative data. PLoS One. (2016) 11(2):e0148937. 10.1371/journal.pone.0148937 26863023 PMC4749276

[21] Niklassen AS Ovesen T Fernandes H Fjaeldstad AW. Danish validation of Sniffin’ Sticks olfactory test for threshold, discrimination, and identification. Laryngoscope. (2018) 128(8):1759–66. 10.1002/lary.27052 29266246

[22] Balungwe P Huart C Matanda R Bisimwa G Mouraux A Rombaux P. Adaptation of the Sniffin’ Sticks test in south-kivu. Eur Ann Otorhinolaryngol Head Neck Dis. (2020) 137(6):467–71. 10.1016/j.anorl.2020.01.012 32044270

[23] Delgado-Losada ML Delgado-Lima AH Bouhaben J. Spanish validation for olfactory function testing using the Sniffin’ Sticks olfactory test: threshold, discrimination, and identification. Brain Sci. (2020) 10(12):943. 10.3390/brainsci10120943 33297359 PMC7762307

[24] Hsu NI Lai JT Shen PH. Development of Taiwan smell identification test: a quick office-based smell screening test for Taiwanese. Am J Rhinol Allergy. (2015) 29(2):e50–4. 10.2500/ajra.2015.29.4174 25785743

[25] Hsieh CH Chen PG Zhou B Lin LJ Lai JT Shen PH. Investigation of normative value of commercialized Taiwan smell identification test. Allergy Rhinol (Providence). (2021) 12:2152656721991525. 10.1177/2152656721991525 33643679 PMC7894691

[26] Kevin SD Govindaraju R Danaee M Shahrizal TA Prepageran N. A preliminary study of the original tibsit and its cultural adaptation in Malaysia. Med J Malaysia. (2021) 76(Suppl 4):3–8. 34558549

[27] Jiang RS Wang JJ Liang KL Shih KH. Validation of the local applicability of the ‘tib’ olfactory test device in the era of COVID-19. J Int Med Res. (2022) 50(1):3000605211069281. 10.1177/03000605211069281 34994243 PMC8743949

[28] Oleszkiewicz A Schriever VA Croy I Hahner A Hummel T. Updated Sniffin’ Sticks normative data based on an extended sample of 9139 subjects. Eur Arch Otorhinolaryngol. (2019) 276(3):719–28. 10.1007/s00405-018-5248-1 30554358 PMC6411676

[29] Mak HWF Chan ETS Yim JSH Lee E Lam DLY Chiang V Validation of the Chinese drug hypersensitivity quality of life questionnaire: role of delabeling. Asia Pac Allergy. (2023) 13(1):3–9. 10.5415/apallergy.0000000000000020 37389094 PMC10166238

[30] Akoglu H. User’s guide to correlation coefficients. Turk J Emerg Med. (2018) 18(3):91–3. 10.1016/j.tjem.2018.08.001 30191186 PMC6107969

[31] R Core Team R. R: A Language and Environment for Statistical Computing. (2013)).

[32] Cho JH Jeong YS Lee YJ Hong SC Yoon JH Kim JK. The Korean version of the Sniffin’ Stick (Kvss) test and its validity in comparison with the cross-cultural smell identification test (Cc-Sit). Auris Nasus Larynx. (2009) 36(3):280–6. 10.1016/j.anl.2008.07.005 18775610

[33] Pinkhardt EH Liu H Ma D Chen J Pachollek A Kunz MS Olfactory screening of Parkinson’s disease patients and healthy subjects in China and Germany: a study of cross-cultural adaptation of the Sniffin’ Sticks 12-identification test. PLoS One. (2019) 14(11):e0224331. 10.1371/journal.pone.0224331 31703081 PMC6839844

[34] Pawankar R Wang JY Wang IJ Thien F Chang YS Latiff AHA Asia Pacific Association of allergy asthma and clinical immunology white paper 2020 on climate change, air pollution, and biodiversity in Asia-Pacific and impact on allergic diseases. Asia Pac Allergy. (2020) 10(1):e11. 10.5415/apallergy.2020.10.e11 32099833 PMC7016319

[35] Lee T-H Leung T-F Wong G Ho M Duque JR Li PH The unmet provision of allergy services in Hong Kong impairs capability for allergy prevention-implications for the Asia Pacific Region. Asian Pac J Allergy Immunol. (2019) 37(1):1–8. 10.12932/ap-250817-0150 29223147

[36] Prepageran N Wang de Y Nair G Maurer M. The Status quo and unmet needs in the management of allergic rhinitis and chronic rhinosinusitis: a Malaysian perspective. Asia Pac Allergy. (2014) 4(3):142–8. 10.5415/apallergy.2014.4.3.142 25097849 PMC4116040

[37] Wang X Zhang N Bo M Holtappels G Zheng M Lou H Diversity of T(H) cytokine profiles in patients with chronic rhinosinusitis: a multicenter study in Europe, Asia, and oceania. J Allergy Clin Immunol. (2016) 138(5):1344–53. 10.1016/j.jaci.2016.05.041 27544740

[38] Shao S Zheng M Wang X Latiff AH Kim DY Wang JY Asia-Pacific survey of physicians’ perceptions and managements of chronic rhinosinusitis. Asian Pac J Allergy Immunol. (In press). 10.12932/AP-130122-1302 35598189

[39] Zheng M Wang X Latiff AHA Shah A Pham DL Kim DY An online survey of clinical practice for allergic rhinitis among the Asia-Pacific representatives. Asian Pac J Allergy Immunol. (In press). 10.12932/AP-310322-1361 36278781

[40] Li PH Pawankar R Thong BY Fok JS Chantaphakul H Hide M Epidemiology, management, and treatment access of hereditary angioedema in the Asia Pacific Region: outcomes from an international survey. J Allergy Clin Immunol Pract. (2023) 11(4):1253–60. 10.1016/j.jaip.2022.12.021 36584968

[41] Li PH Pawankar R Thong BYH Mak HWF Chan G Chung WH Disparities and inequalities of penicillin allergy in the Asia-Pacific Region. Allergy. (2023) 78(9):2529–32. 10.1111/all.15725 36951669

[42] Attems J Walker L Jellinger KA. Olfaction and aging: a mini-review. Gerontology. (2015) 61(6):485–90. 10.1159/000381619 25968962

[43] Evans WJ Cui L Starr A. Olfactory event-related potentials in normal human subjects: effects of age and gender. Electroencephalogr Clin Neurophysiol. (1995) 95(4):293–301. 10.1016/0013-4694(95)00055-4 8529560

[44] Mullol J Alobid I Marino-Sanchez F Quinto L de Haro J Bernal-Sprekelsen M Furthering the understanding of olfaction, prevalence of loss of smell and risk factors: a population-based survey (olfacat study). BMJ Open. (2012) 2(6):e001256. 10.1136/bmjopen-2012-001256 23135536 PMC3533119

[45] Lee K Choi IH Lee SH Kim TH. Association between subjective olfactory dysfunction and female hormone-related factors in South Korea. Sci Rep. (2019) 9(1):20007. 10.1038/s41598-019-56565-x 31882785 PMC6934502

