# Supplementary material for: Clinical utility of and correlation between Sniffin' Sticks and TIB smell identification test (TIBSIT) among Hong Kong Chinese with or without chronic rhinosinusitis
Source: Front Allergy. 2024 Jan 24;5:1292342. doi: 10.3389/falgy.2024.1292342 (PMC10847303; doi:10.3389/falgy.2024.1292342)
Supplement: Supplementary file 1 [file Table1.docx]

## Supplementary Table 1 Multivariate linear regression for predictors of olfactory test scores

|  | **Presence of CRS** | | **Age** | | **Female sex** | |
| --- | --- | --- | --- | --- | --- | --- |
|  | **p-value** | **β (standardised coefficient)** | **p-value** | **β (standardised coefficient)** | **p-value** | **β (standardised coefficient)** |
| T score (SST) | ***<0.001** | -0.746 | 0.099 | -0.178 | ***0.039** | 0.225 |
| D score (SST) | ***<0.001** | -0.694 | ***0.003** | -0.353 | — | — |
| I score (SST) | ***<0.001** | -0.624 | 0.346 | -0.135 | — | — |
| TDI score (SST) | ***<0.001** | -0.790 | ***0.006** | -0.274 | — | — |
| TIBSIT score | ***<0.001** | -0.858 | ***0.001** | -0.301 | — | — |

Bold denotes statistical significance.

CRS, chronic rhinosinusitis; TIBSIT, TIB Smell Identification Test.

T, threshold; D, discrimination; I, identification; TDI, composite of T, D and I scores.

**Supplementary Figure 1 Olfactory test scores stratified by sex**

TIBSIT, TIB Smell Identification Test. T, threshold; D, discrimination; I, identification; TDI, composite of T, D and I scores.
