# Supplementary figures and images for: Clinical utility of and correlation between Sniffin' Sticks and TIB smell identification test (TIBSIT) among Hong Kong Chinese with or without chronic rhinosinusitis
Source: Front Allergy. 2024 Jan 24;5:1292342. doi: 10.3389/falgy.2024.1292342 (PMC10847303; doi:10.3389/falgy.2024.1292342)

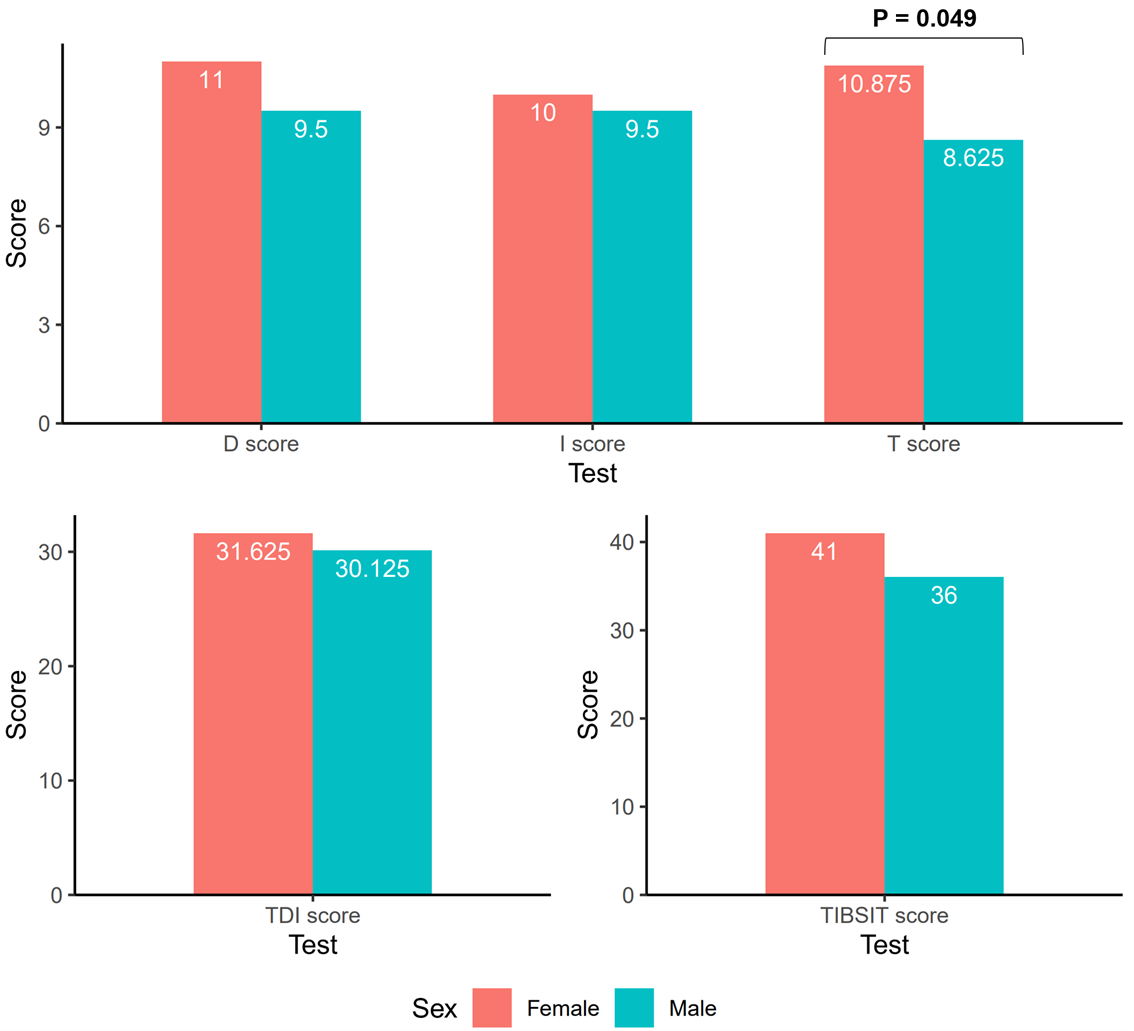

Supplement: Supplementary file 2 [file Image1.tif]
